# Supplementary material for: Unbiased recording and identification of thymic cellular interactomes using synthetic Notch receptors
Source: Nat Commun. 2026 Mar 9;17:3708. doi: 10.1038/s41467-026-70225-5 (PMC13102935; doi:10.1038/s41467-026-70225-5)
Supplement: Supplementary file 1 — Supplementary Information [file 41467_2026_70225_MOESM1_ESM.pdf]

Unbiased recording and identification of thymic cellular interactomes using synthetic Notch receptors.

Raúl Sánchez-Lanzas[1], Amanda Jiménez-Pompa[1], Elise Smith[2], Nital Sumaria[3], Justin Barclay[1], Foteini Kalampalika[1], Daniel Pennington[3], Mirjana Efremova[2*], Miguel Ganuza[1*].

Affiliations: [1]Centre for Haemato-Oncology – Barts Cancer Institute (BCI), Queen Mary University of London (QMUL); London, EC1M 6BQ, United Kingdom. [2]Center for Cancer Genomics and Computational Biology – BCI, QMUL; London, EC1M 6BQ, United Kingdom. [3]Centre for Immunobiology – Blizzard Institute, QMUL; London, 4 Newark St, London E1 2AT, United Kingdom.

SUPPLEMENTARY MATERIALS

Supplementary Figures.

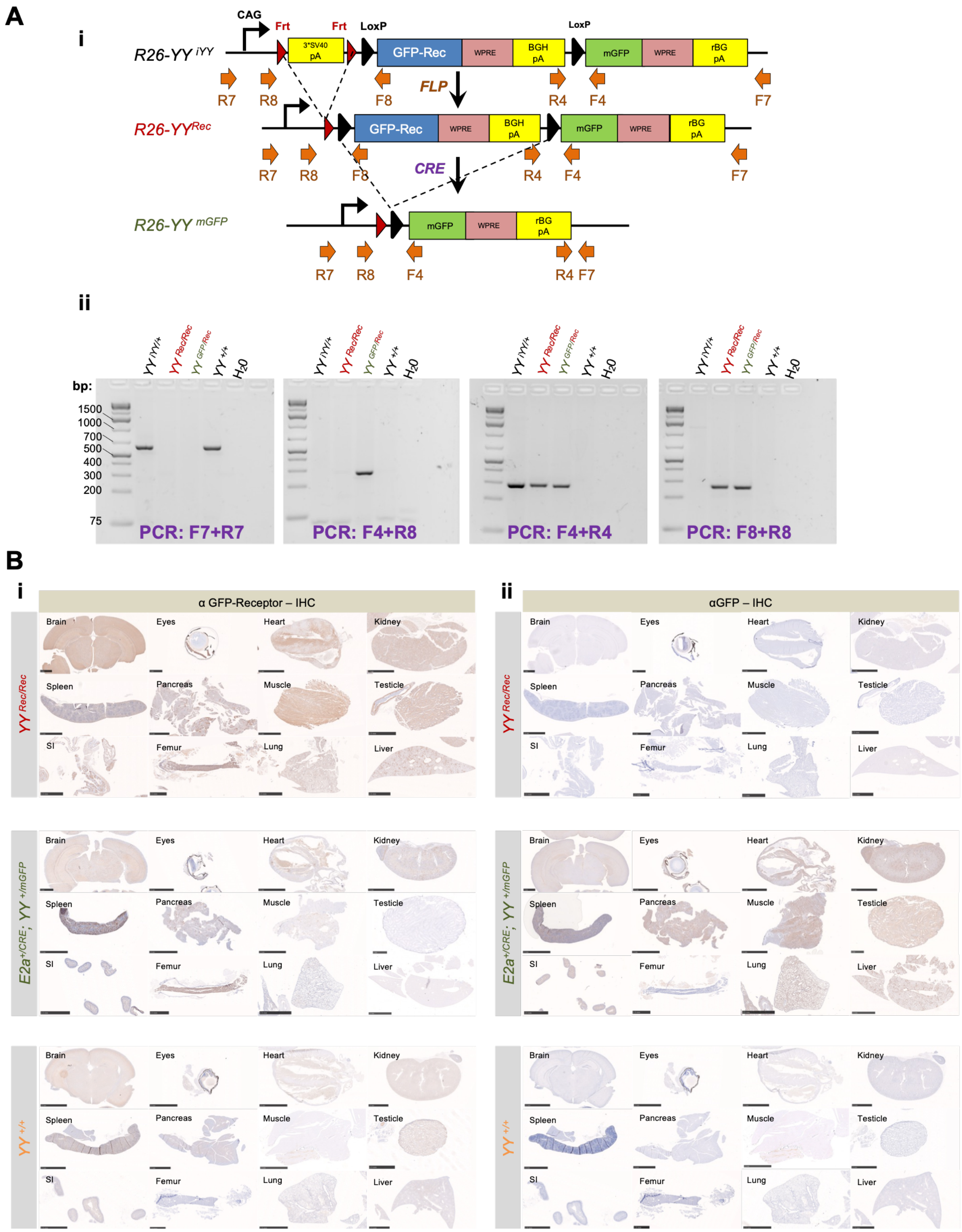

Figure S1. GFP-Receptor is ubiquitously expressed in ***YY*** $^{REC/REC}$ **receiver mice and mGFP only expressed following CRE-driven recombination. A.** Genotyping strategy for the Yin&Yang alleles. **Ai.** Schematic of the Yin&Yang alleles which includes:

unrecombined $R26^{frtSTOPfrt\text{-}lox\text{-}GFP\text{-}Rec\text{-}lox\text{-}mGFP}$ (YY^{YY}), $R26\text{-}YY^{lox\text{-}GFP\text{-}Rec\text{-}lox\text{-}mGFP}$ (YY^{Rec}) and YY^{mGFP}. Location of genotyping primers (F4, R4, F8, R8, F7 and R7) is indicated on each allele. **Aii.** 2% Agarose gels for genotyping PCRs. F4+R4, F8+R8, and F4+R8 are shown for tissues isolated from $YY^{+/YY}$ unrecombined mice, $YY^{REC/REC}$ receiver mice and $YY^{mGFP/REC}$ mice (carrying both alleles). Uncropped image in SF7. **Bi.** αMYC-TAG IHC of representative areas of multiple tissues obtained from 8 weeks old control $YY^{+/+}$wild-type mice, $YY^{REC/REC}$ receiver mice and $E2a^{+/CRE}$ $YY^{+/mGFP}$ sender mice, showing that the GFP-REC (detected by the αMYC-tag antibody) is ubiquitously expressed in every analyzed tissue in the mouse, converting every cell into a potential receiver. **Bii.** αGFP IHC of representative areas of multiple tissues from the same mice. Importantly, GFP is not detected in any tissue of receiver mice, confirming the lack of GFP^+ sender cells in the absence of CRE recombinase, validating the robustness of the system. ($E2a^{+/CRE}$ $YY^{+/mGFP}$ sender mice showed widespread GFP expression demonstrating that CRE expression is able to drive mGFP expression in every tissue, allowing the inducible and controlled presence of sender cells. Scale bar 2.5 mm

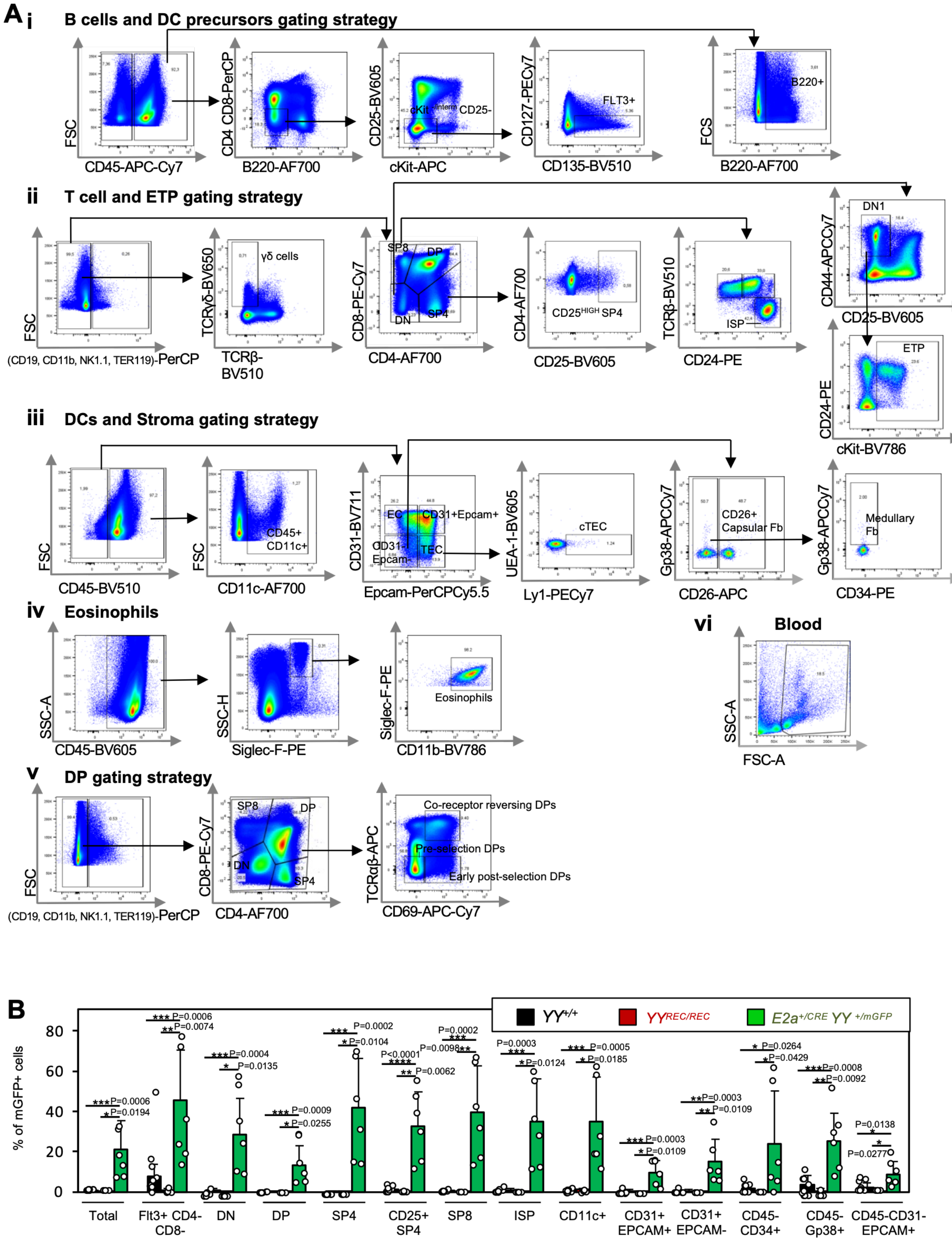

Figure S2. Analysis of GFP expression *in* Yin&Yang thymuses. Flow cytometry gating strategies. A. Representative flow cytometry gating strategy for the different staining combinations employed to identify cellular populations in the thymuses, including

B220^{+} cells and DC precursors **(Ai)**, T cells and ETPs **(Aii)**, CD11c+ cells (enriched in DCs) and stromal cells **(Aiii)**, eosinophils **(Aiv)** and thymic DP subpopulations (Av); and peripheral blood populations (Avi). **B.** Flow cytometry analyses on the frequency of GFP^{+} cells across thymic populations in $YY^{REC/REC}$ receiver mice (n=4), $E2a^{+/CRE}$ $YY^{+/mGFP}$ sender mice (n=6) and $YY^{+/+}$ wild type (n=9), showing that GFP expression is only detected upon CRE activity in the thymuses of Yin&Yang mice (five independent experiments). Analyzed populations included: FLT3+ CD4$^{–}$CD8$^{–}$, DN, DP, SP4, SP8, CD25^{+}SP4, ISPs (CD4$^{–}$CD8^{+}CD24^{+}TCRβ$^{–}$), CD45^{+}CD11c^{+} (enriched in DCs), endothelial cells (ECs, CD45$^{–}$CD31^{+}EPCAM$^{–}$), CD45$^{–}$CD31^{+}EPCAM^{+}, CD45$^{–}$CD34^{+}, CD45$^{–}$Gp38^{+} and thymic epithelial cells (TECs, CD45$^{–}$CD31$^{–}$EPCAM^{+}). Means and standard deviations are indicated. Each individual point represents an independent mouse. Unpaired two-tailed t-test. ****p <0.0001, ***p <0.001, **p <0.01, *p <0.05.

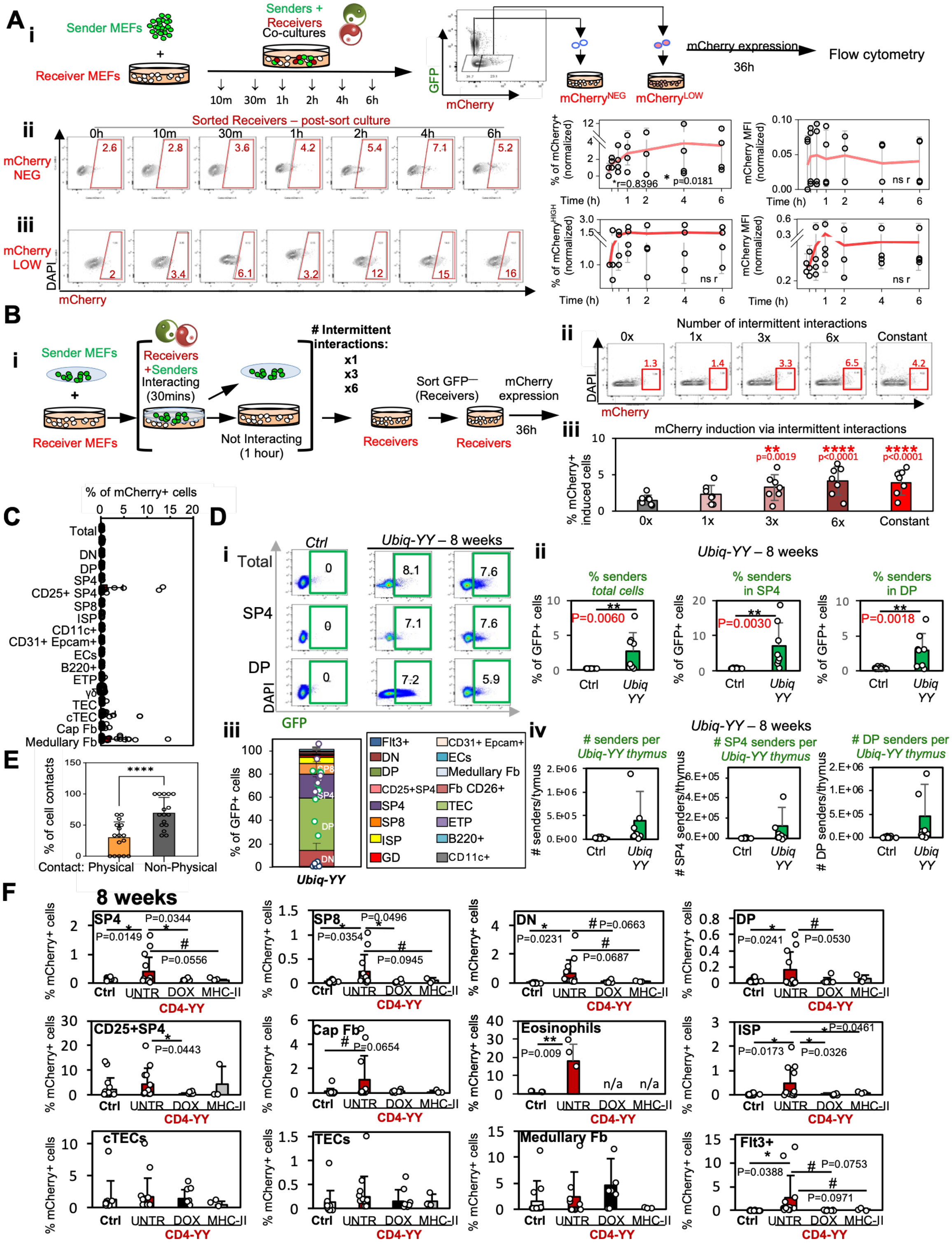

Figure S3. The Yin&Yang system can record repetitive intermittent cell interactions that lasted <1 hour. Characterization of senders in *Ubiq-YY* mice and the SP4/DP interactome. A. H2B-mCherry expression from the TRE^{Cherry} allele records cell contacts

of ≥2 hours. **Ai.** Experimental schematic. Sender and receiver MEFs were co-cultured for 10 min, 30min, 1h, 2h, 4h and 6h. Receiver cells were then sorted from these co-cultures as mCherry^{NEG} and mCherry^{LOW} and replated as mono-cultures for 36h additional hours to allow mCherry expression post-cell contact. The increase in mCherry expression (MFI) was evaluated according to the time of co-culture. Representative flow cytometry plots of sorted receiver MEFs. mCherry^{NEG} **(Aii)** and mCherry^{LOW} (**Aiii)** sorted fractions. **Aii.** % of mCherry^{+} cells and mCherry MFI for sorted mCherry^{NEG} according to the time of initial co-culture **(**n=4 from 2 different experiments). **Aiii**, % of mCherry^{HIGH} cells and mCherry MFI for sorted mCherry^{LOW} according to the time of initial co-culture (n=4 from 2 different experiments) [Pearson r; p (two tailed) are shown]. **B.** Three repetitive intermittent cell interactions of 30 minutes results in detectable recording of cell interactions. Interacting receiver cells get labeled as H2B-mCherry^{+}. **Bi.** Experimental schematic. Senders MEFs (grown on cover slips) were put in contact with receiver MEFs for 30 mins. Coverslips enabled timely removal of receivers. After a one-hour non-interacting gap, interaction was repeated or not. The 30 minutes interaction + 1hour gap was repeated x1, x3 or 6x times. Receiver cells were FACS-sorted to eliminate any sender that could have been transferred to the coverslip. Sorted receivers were cultured for additional 36 hours to enable mCherry expression. **Bii.** Representative flow cytometry plots of sorted receivers 36 hours post-culture. **Biii.** Quantification of mCherry^{+} induction following repetitive intermittent interactions. [n=7 biological replicates, results pooled from two independent experiments. Two-way ANOVA (F (4, 24)=10.23. Uncorrected Fisher's LDS for individual time points]. **C.** Frequencies of mCherry^{+} cells across thymic populations in *YY$^{REC/REC}$ TRE$^{Cherry/Cherry}$* Cre-negative Control mice (n=28) were analyzed by flow cytometry demonstrating that mCherry^{+} expressing background cells are equally distributed among thymus cells and ~0.1% of total cells. Background in Fb and Tregs was higher. **D.** Analysis of the cellular identity of senders in *Ubiq-CreERT2$^{+/Cre-ERT2}$ YY$^{REC/REC}$TRE$^{Cherry/Cherry}$* (*Ubiq-YY*) thymuses. TAM-treated *Ubiq-YY* mice (n=8) were analyzed via flow cytometry. TAM was administered for 11 days (2mg/day) and analyzed 15 days post-initiation of treatment. **Di.** Flow cytometry plots for the presence of GFP^{+} senders in total, SP4 and DP cells. **Dii.** Quantification of % of senders within total, SP4 and DP cells. **Diii.** Distribution of cellular identities among GFP^{+} sender cells in *Ubiq-YY* thymuses. **Div.** Quantification of absolute

numbers of sender cells within total, SP4 and DP cells. **E.** Related to Fig. 3Biii. Confocal micrographs of TAM-treated *Ubiq-YY* thymuses were analyzed for the cellular proximity among $mGFP^+$ sender cells and $mCherry^+$ labeled cells to determine if $mGFP^+$ senders are in physical cell-to-cell contact to interacting $mCherry^+$ cells at the time of analysis, which it is reflective on both cellular mobility and the ability of the *YY* system to record more transient interactions. % of $mCherry^+$ cells in physical contact and no-physical contact are shown (n=62 cells from 5 mice analyzed in 16 independent experiments; Unpaired t test). **F.** Related to Fig. 5C. Flow cytometry analysis of the cellular interactome that supports SP4 cells in 6 and 8 weeks old mice. % of $mCherry^+$ cells (recorded interactions) was evaluated within each indicated cell population for *CD4-YY* (untreated, UNTR, n=12; four independent experiments) thymuses and compared to those in Ctrl (n=15, from 6 independent experiments) and treated *CD4-YY* mice (DOX, n=8, four independent experiments; αMHC-II, n=3, two independent experiments). **A-F.** Means and standard deviations are indicated. Each individual point represents an independent culture, analyzed cell contact or mouse. Pearson correlation is shown as r. **Dii**, **Div** and **F**, unpaired two-tailed t-test. ns: not statistically significant. ****p <0.0001, ***p <0.001, **p <0.01, *p <0.05, # p <0.1.

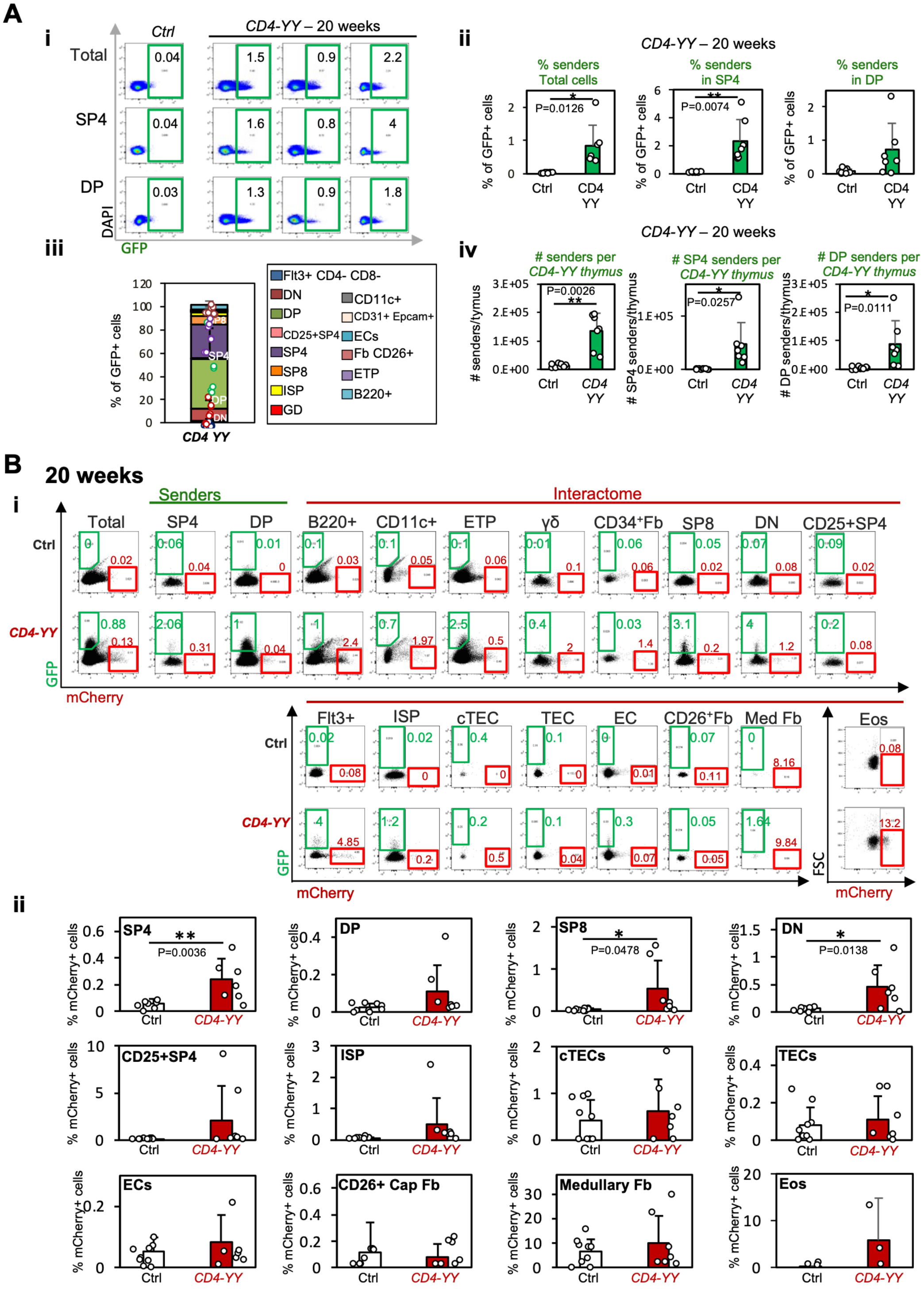

Figure S4. Identification of the cellular interactome that supports SP4 and DP cells in 20 weeks old mice by flow cytometry analysis. A. Analysis of the cellular identity of senders in 20 weeks old *CD4-YY* thymuses (n=7, three independent experiments) were analyzed via flow cytometry. **Ai.** Flow cytometry plots for GFP+ senders in total, SP4 and

DP cells. **Aii.** Quantification of % of senders within total, SP4 and DP cells. **Aiii.** Distribution of cellular identities among GFP^+ sender cells. **Aiv.** Quantification of absolute numbers of sender cells within total, SP4 and DP cells. **B.** Flow cytometry analysis of the cellular interactome that supports SP4 cells at 20 weeks. Related to Fig. 7Ai. $mCherry^+$ enrichment (recorded interactions) within each indicated thymic population in 20 weeks old *CD4-YY* thymuses (n=7) and $YY^{REC/REC}$ $TRE^{Cherry/Cherry}$ CRE-negative control thymuses (Ctrl) (n=9) from three independent experiments. **Bi.** Representative flow cytometry plots. **Bii.** Quantification of % of $mCherry^+$ cells. Means and standard deviations are depicted. Each individual point represents an independent mouse. **Aii, Aiv** and **Bii**, unpaired two-tailed t-test. **p <0.01, *p <0.05.

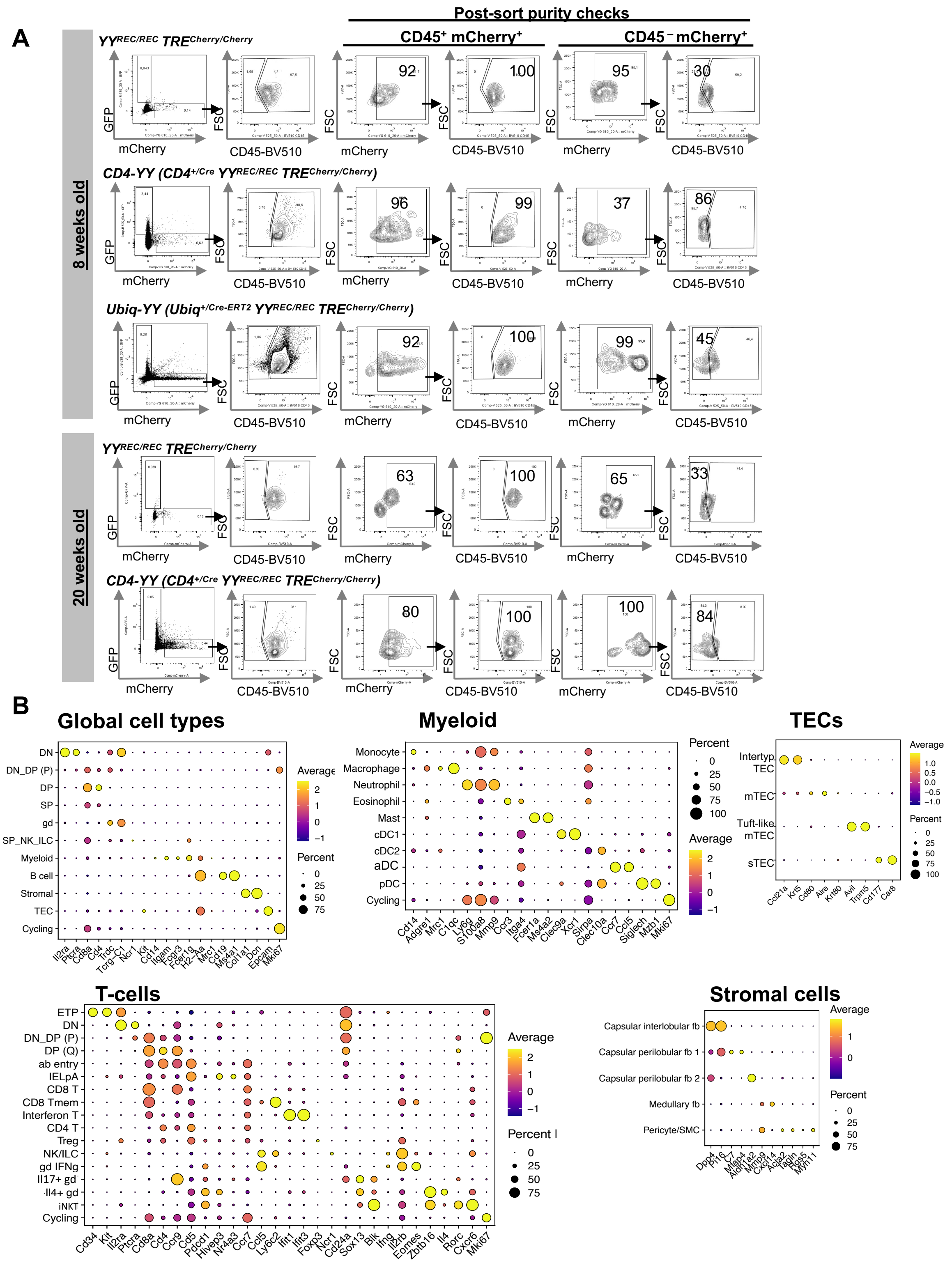

Figure S5. Markers employed in scRNAseq analysis for the identification of cellular populations within the cellular interactome that supports thymic SP4 and DP cells. **A.** Related to Figs. 6, 7Aii, 7Bii & S6. Post-sort purity checks were run for mCherry^{+} cells

sorted from the thymuses of cohorts of *YY*$^{REC/REC}$ *TRE*$^{Cherry/Cherry}$ CRE-negative Ctrl mice, *CD4-YY* mice and TAM-treated *Ubiq-YY* mice. **B.** Dot plots with marker genes for populations identified by scRNAseq. Supplementary table 1 shows the top 50 markers for all cell types.

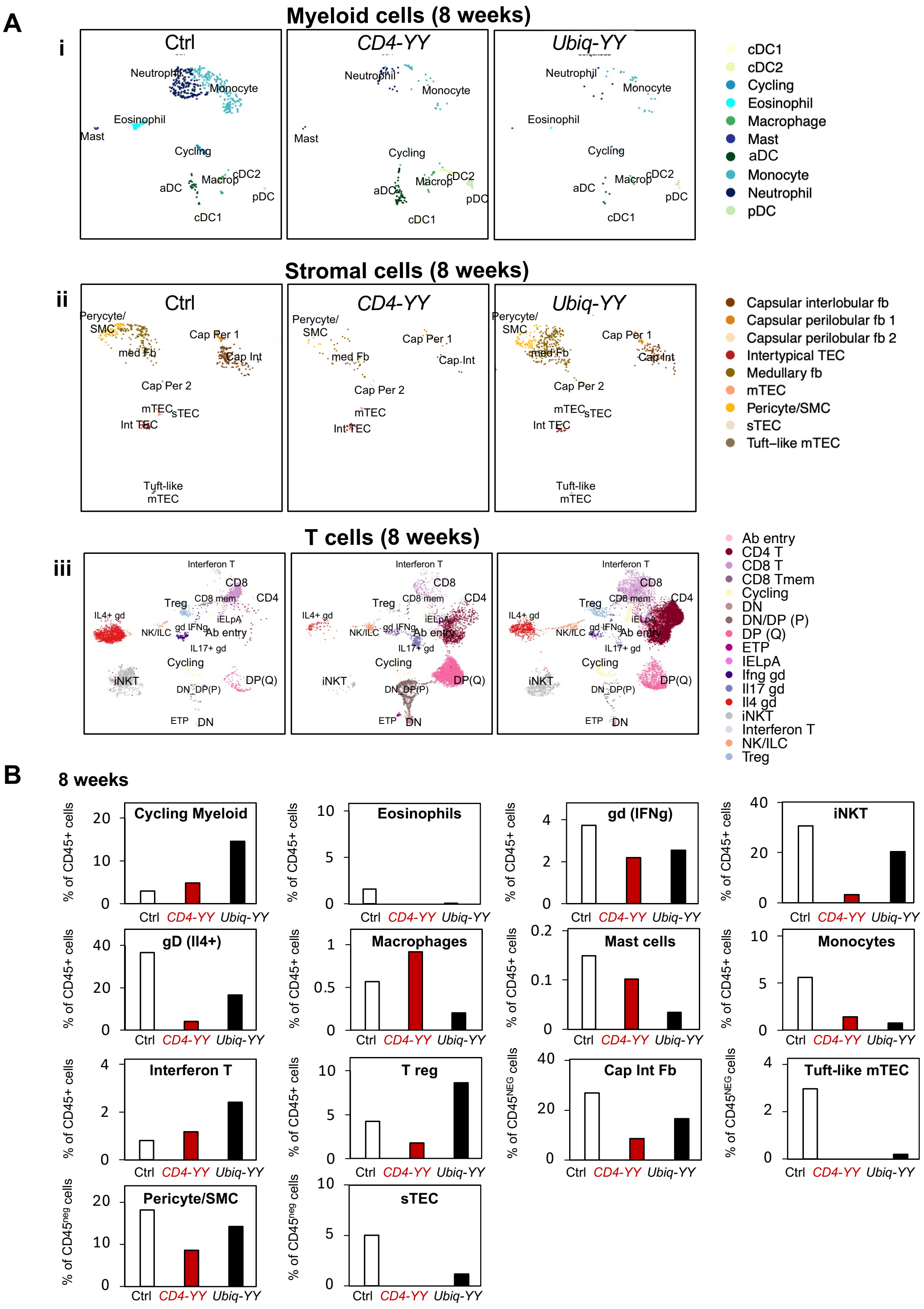
A
Myeloid cells (8 weeks)
i
Ctrl
CD4-YY
Ubiq-YY
Neutrophil
Monocyte
Eosinophil
Mast
Cycling
aDC
Macrop
cDC2
pDC
cDC1
cDC1
cDC2
Cycling
Eosinophil
Macrophage
Mast
aDC
Monocyte
Neutrophil
pDC
Stromal cells (8 weeks)
ii
Perycyte/ SMC
Cap Per 1
med Fb
Cap Int
Cap Per 2
mTEC
sTEC
Int TEC
Tuft-like mTEC
Capsular interlobular fb
Capsular perilobular fb 1
Capsular perilobular fb 2
Intertypical TEC
Medullary fb
mTEC
Pericyte/SMC
sTEC
Tuft–like mTEC
T cells (8 weeks)
iii
Interferon T
CD8
CD8 mem
Treg
CD4
IL4+ gd
iELpA
NK/ILC
gd IFNg
Ab entry
IL17+ gd
Cycling
iNKT
DP(Q)
DN
DP(P)
ETP
Ab entry
CD4 T
CD8 T
CD8 Tmem
Cycling
DN
DN/DP (P)
DP (Q)
ETP
IELpA
Ifng gd
Il17 gd
Il4 gd
iNKT
Interferon T
NK/ILC
Treg
B
8 weeks
Cycling Myeloid
Eosinophils
gd (IFNg)
iNKT
gD (Il4+)
Macrophages
Mast cells
Monocytes
Interferon T
T reg
Cap Int Fb
Tuft-like mTEC
Pericyte/SMC
sTEC
% of CD45+ cells
% of CD45NEG cells
% of CD45neg cells
Ctrl
CD4-YY
Ubiq-YY

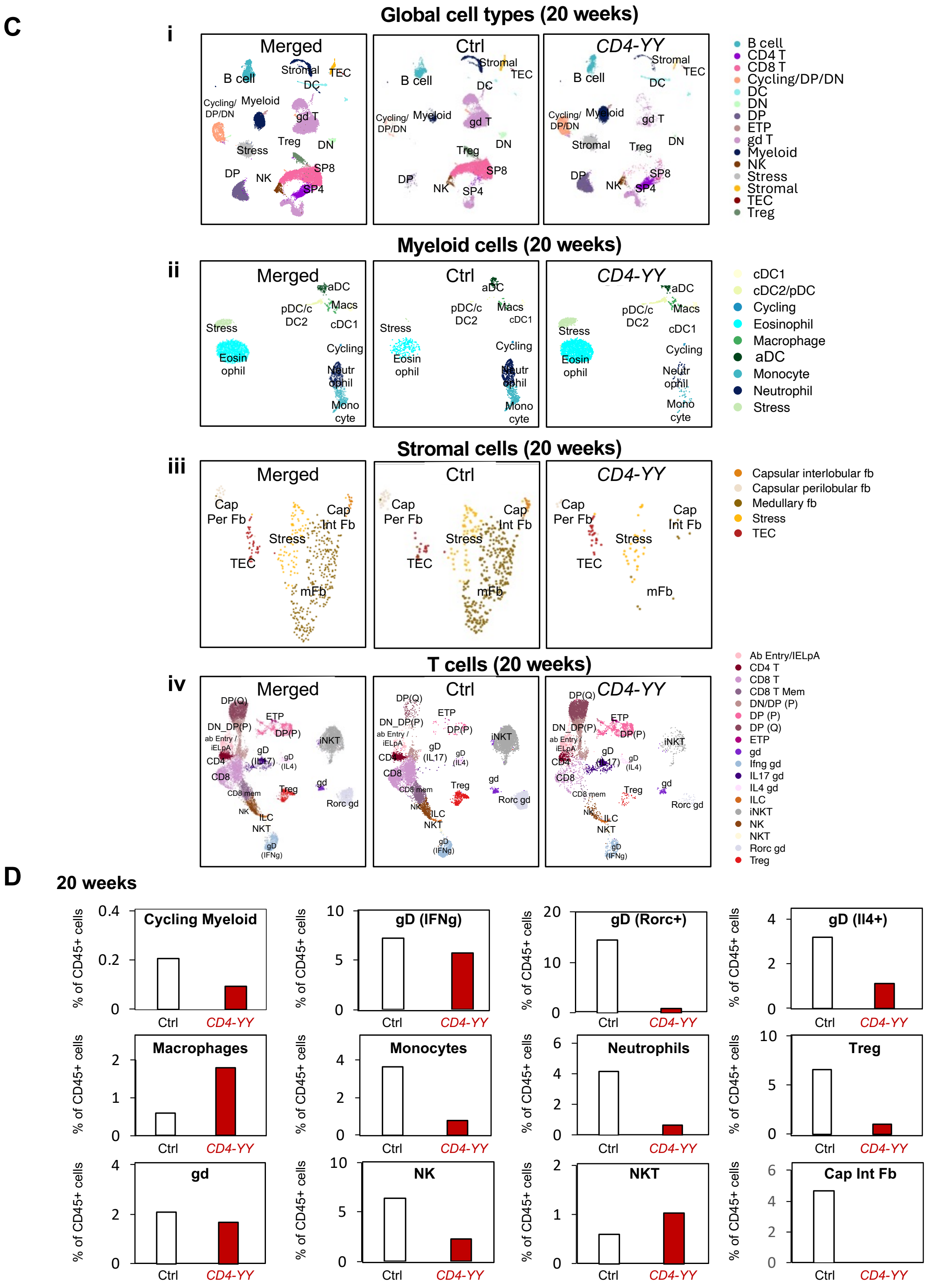

Figure S6. Identification of the cellular interactome that supports thymic SP4 and DP cells by scRNAseq analysis. The cellular interactomes of SP4 and DP cells were defined

by transcriptional profiling of mCherry^{+} cells isolated from *CD4-YY* thymuses (pooled from 8 thymuses of 8 weeks old mice and 11 thymuses of 20 weeks old mice), *YY$^{REC/REC}$ TRE$^{Cherry/Cherry}$* CRE-negative Control thymuses (n=12 at 8 weeks old and n=18 at 20 weeks old) and *Ubiq-YY* thymuses (pooled from 13 thymuses of 8 weeks old mice)**.** UMAP representations of cell cluster annotation of the cellular interactomes (within mCherry^{+} sorted cells) that supports SP4 and DP cells in the thymus of 8 weeks **(A)** and 20 weeks old mice **(C)**. The frequencies of each cell cluster were calculated within mCherry^{+}CD45^{+} and mCherry^{+}CD45^{-} cells and are shown at 8- **(B)** and 20-weeks old **(D)** thymuses. Frequencies of cell clusters not included in Figs. 6 and 7Aii are shown.

A

Uncropped gel - Related to SF1Aii

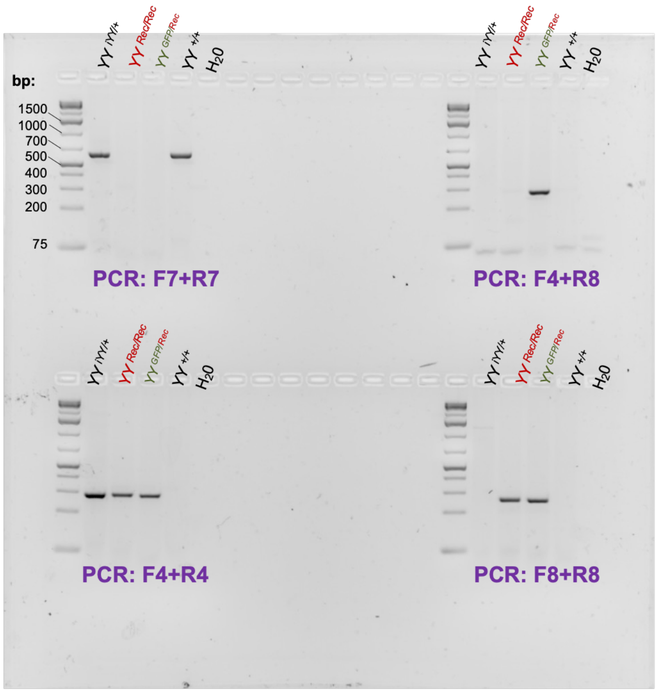

B

Uncropped images - Related to Fig. 1Cii

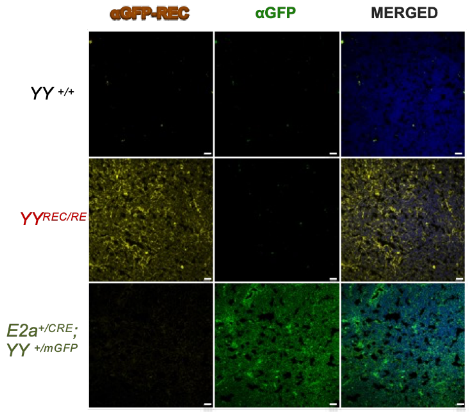

C

Uncropped images - Related to Fig. 2Aiv

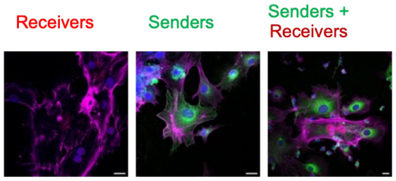

Figure S7. Uncropped images. A. Related to Figure S1Aii. **B.** Related to Figure 1Cii. **C.** Related to Figure 2Aiv.

Supplementary Tables.

Supplementary tables are provided as excel files

Table S1. Top 50 DEG for each cluster

Source data is provided as an excel file
