# Supplementary material for: Unbiased recording and identification of thymic cellular interactomes using synthetic Notch receptors
Source: Nat Commun. 2026 Mar 9;17:3708. doi: 10.1038/s41467-026-70225-5 (PMC13102935; doi:10.1038/s41467-026-70225-5)
Supplement: Supplementary file 3 — Reporting Summary [file 41467_2026_70225_MOESM3_ESM.pdf]

nature portfolio

Corresponding author(s): Mirjana Efemova and Miguel Ganuza

Last updated by author(s): Feb 11, 2026

Reporting Summary

Nature Portfolio wishes to improve the reproducibility of the work that we publish. This form provides structure for consistency and transparency in reporting. For further information on Nature Portfolio policies, see our Editorial Policies and the Editorial Policy Checklist.

Statistics

For all statistical analyses, confirm that the following items are present in the figure legend, table legend, main text, or Methods section.

n/a	Confirmed	
☐	☒	The exact sample size (n) for each experimental group/condition, given as a discrete number and unit of measurement
☐	☒	A statement on whether measurements were taken from distinct samples or whether the same sample was measured repeatedly
☐	☒	The statistical test(s) used AND whether they are one- or two-sided<br>*Only common tests should be described solely by name; describe more complex techniques in the Methods section.*
☒	☐	A description of all covariates tested
☐	☒	A description of any assumptions or corrections, such as tests of normality and adjustment for multiple comparisons
☐	☒	A full description of the statistical parameters including central tendency (e.g. means) or other basic estimates (e.g. regression coefficient) AND variation (e.g. standard deviation) or associated estimates of uncertainty (e.g. confidence intervals)
☐	☒	For null hypothesis testing, the test statistic (e.g. F, t, r) with confidence intervals, effect sizes, degrees of freedom and P value noted<br>*Give P values as exact values whenever suitable.*
☒	☐	For Bayesian analysis, information on the choice of priors and Markov chain Monte Carlo settings
☒	☐	For hierarchical and complex designs, identification of the appropriate level for tests and full reporting of outcomes
☐	☒	Estimates of effect sizes (e.g. Cohen's d, Pearson's r), indicating how they were calculated

Our web collection on statistics for biologists contains articles on many of the points above.

Software and code

Policy information about availability of computer code

Data collection	No private code was used for data collection.
Data analysis	- Image J 1.54f was employed to prepare all the figures containing immunofluorescence and immunohistochemistry experiments.<br>- FlowJo version 10.10 was used to analyze flow cytometry data.<br>- GraphPad Prism 9 was used for statistical analysis.<br>- 10x Genomics' Cell Ranger (v2.0) was used to pre-process scRNA-seq data.<br>- Seurat v4 was used for downstream analysis of the scRNA-seq data. CellPhoneDB (v3.1.0) was used for cell-cell communication analysis.<br>- NDP.view2Plus (Hamamatsu) was used to image and analyze the scanned immunohistochemistry slides.

For manuscripts utilizing custom algorithms or software that are central to the research but not yet described in published literature, software must be made available to editors and reviewers. We strongly encourage code deposition in a community repository (e.g. GitHub). See the Nature Portfolio guidelines for submitting code & software for further information.

Data

Policy information about availability of data

All manuscripts must include a data availability statement. This statement should provide the following information, where applicable:

- Accession codes, unique identifiers, or web links for publicly available datasets
- A description of any restrictions on data availability
- For clinical datasets or third party data, please ensure that the statement adheres to our policy

Data and material availability: All unique reagents generated in this study (Yin&Yang mouse strains and immortalized cell lines) are available with a completed Materials Transfer Agreement. Yin&Yang mice will be deposited in mouse repositories to enable easier access following publication. scRNAseq data generated in this study have been deposited in the NCBI-SRA database under BioProject ID: PRJNA1276306. [https://www.ncbi.nlm.nih.gov/bioproject/?term=PRJNA1276306]. Source data generated in this study are provided in the Supplementary Information/Source Data file. Additional underlying data supporting the findings of this study, including raw flow cytometry (FCS) files are available from the corresponding authors upon reasonable request. Requests for access should be submitted by email to the corresponding authors and include a brief description of the intended use. Access will be granted for academic, non-commercial research purposes, subject to appropriate data use and not redistributed.

Research involving human participants, their data, or biological material

Policy information about studies with human participants or human data. See also policy information about sex, gender (identity/presentation), and sexual orientation and race, ethnicity and racism.

Reporting on sex and gender	*Use the terms sex (biological attribute) and gender (shaped by social and cultural circumstances) carefully in order to avoid confusing both terms. Indicate if findings apply to only one sex or gender; describe whether sex and gender were considered in study design; whether sex and/or gender was determined based on self-reporting or assigned and methods used.*<br>*Provide in the source data disaggregated sex and gender data, where this information has been collected, and if consent has been obtained for sharing of individual-level data; provide overall numbers in this Reporting Summary. Please state if this information has not been collected.*<br>*Report sex- and gender-based analyses where performed, justify reasons for lack of sex- and gender-based analysis.*
Reporting on race, ethnicity, or other socially relevant groupings	*Please specify the socially constructed or socially relevant categorization variable(s) used in your manuscript and explain why they were used. Please note that such variables should not be used as proxies for other socially constructed/relevant variables (for example, race or ethnicity should not be used as a proxy for socioeconomic status).*<br>*Provide clear definitions of the relevant terms used, how they were provided (by the participants/respondents, the researchers, or third parties), and the method(s) used to classify people into the different categories (e.g. self-report, census or administrative data, social media data, etc.)*<br>*Please provide details about how you controlled for confounding variables in your analyses.*
Population characteristics	*Describe the covariate-relevant population characteristics of the human research participants (e.g. age, genotypic information, past and current diagnosis and treatment categories). If you filled out the behavioural & social sciences study design questions and have nothing to add here, write "See above."*
Recruitment	*Describe how participants were recruited. Outline any potential self-selection bias or other biases that may be present and how these are likely to impact results.*
Ethics oversight	*Identify the organization(s) that approved the study protocol.*

Note that full information on the approval of the study protocol must also be provided in the manuscript.

Field-specific reporting

Please select the one below that is the best fit for your research. If you are not sure, read the appropriate sections before making your selection.

[x] Life sciences [ ] Behavioural & social sciences [ ] Ecological, evolutionary & environmental sciences

For a reference copy of the document with all sections, see nature.com/documents/nr-reporting-summary-flat.pdf

Life sciences study design

All studies must disclose on these points even when the disclosure is negative.

Sample size	No statistical method was used to predetermine sample size.
Data exclusions	Data exclusion was only applied in the case of the thymus analysis by flow cytometry presented in Figs. 4-5, 7Ai, S3F & S4Aii where animals non-carrying sender cells were removed from the analysis.
Replication	Figure 1C: Images were acquired over three experiments from sections (n=3 per mouse and experiment) obtained from thein thymuses of of YY+/+wild-type mice(n=3), YYREC/REC Receiver mice (n=3) and E2a+/CRE YY+/mGFP Sender mice (n=2).<br>Figure 1Dii: Each individual point represents an independent mouse. R26-YY +/+ (n=4 for peripheral blood from 1 independent experiment and n=9 for thymus analysis from 5 independent experiments); R26-YYRec/Rec (n=5 for peripheral blood from 1 independent experiment and

n=4 for thymus analysis from 2 independent experiments); and E2a+/CRE R26-YY+/mGFP (n=6 for peripheral blood from 1 independent experiment and n=6 for thymus analysis from 3 independent experiments).
Figure 2Aiii: 5 independent experiments. Yang receivers - R26-YYRec/Rec; TRECherry/Cherry (n=14); Yin senders - E2a+/CRE;R26-YY+/mGFP (n=10); co-cultures of Yin+Yang cells (n=32).
Figure 2Bii: for t=6 hours, n=8; for t=12, 24, 36, 48 and 72 hours, n=15 from 3 independent experiments.
Figure 2Biii: for t=6 hours, n=8; for t=12, 24, 36, 48 and 72 hours, n=15 from 3 independent experiments.
Figure 2Cii: n=4 from 3 independent experiments.
Figure 3Bii: Each individual dot represents an individual mouse. R26-YYRec/Rec; TRECherry/Cherry Control mice (Ctrl, n=16), and treated iUbiquitin-YY (n=11), acquired over 9 and 5 independent experiments, respectively.
Figure 4Bi: Ctrl mice, n=7 from 7 independent experiments and CD4-YY thymuses, n=20 from 6 independent experiments.
Figure 4Bii: Ctrl mice, n=7 from 7 independent experiments and CD4-YY thymuses, n=20 from 6 independent experiments.
Figure 4Ci: CD4-YY thymuses, n=16 from 6 independent experiments.
Figure 4Cii: CD4-YY thymuses, n=7 from 1 independent experiment.
Figure 5C: 6-8 weeks old CD4-YY mice, n=12 from 4 independent experiments; YREC/RECTRECherry/Cherry CRE-negative control mice (Ctrl), n=15 from 6 independent experiments; 4 weeks old CD4-YY mice treated with doxycycline, n=8 from 4 different experiments, and CD4-YY mice treated with MHC-II blocking antibody, n=3 from 2 different experiments.
Figure 6: 1 independent experiment. Thymuses from 8-9 weeks old CD4-YY mice were pooled from n=8 thymuses, TAM-treated Ubiq-YY mice were pooled from n=13 thymuses, and untreated Ctrl mice were pooled from n=12 thymuses.
Figure 7Ai: 20 weeks old CD4-YY mice, n=7 from 3 independent experiments and YYREC/REC TRECherry/Cherry CRE-negative control mice (Ctrl), n=9 from 3 different experiments.
Figure 7Aii: Thymuses from 20 weeks old CD4-YY thymuses were pooled from n=11 thymuses from 1 independent experiment and Ctrl mice were pooled from n=18 thymuses from 2 independent experiments.
Figure 7Bi: 6-8 weeks old CD4-YY mice, n=12 from 4 independent experiments; 20 weeks old CD4-YY mice, n=7 from 3 independent experiments.
Figure 7Bii: 1 independent experiment. Thymuses from 8-9 weeks old CD4-YY mice were pooled from n=8 thymuses, and thymuses from 20 weeks old CD4-YY were pooled from n=11 thymuses.
Supplemental Figure 2B: R26-YYRec/Rec (n=4 from 2 independent experiments), E2a+/CRE; R26-YY+/mGFP (n=6, from 3 independent experiments) and R26-YY +/+ (n=9, from 5 independent experiments).
Supplemental Figure 3Aii: n=4 from 2 different experiments.
Supplemental Figure 3Aiii: n=4 from 2 different experiments.
Supplemental Figure 3Biii: n=7 from 2 different experiments.
Supplemental Figure 3C: n=28 from 15 different experiments.
Supplemental Figure 3Dii: TAM-treated Ubiq-YY mice, n=8 from 6 different experiments, Control mice, n=10 from 3 different experiments.
Supplemental Figure 3Diii: TAM-treated Ubiq-YY mice, n=8 from 6 different experiments.
Supplemental Figure 3Div: TAM-treated Ubiq-YY mice, n=8 from 6 different experiments, Control mice, n=10 from 3 different experiments.
Supplemental Figure 3E: n=62 cells from 5 mice analyzed in 16 independent experiments.
Supplemental Figure 3F: 6-8 weeks old CD4-YY mice, n=12 from 4 independent experiments; YREC/RECTRECherry/Cherry CRE-negative control mice (Ctrl), n=15 from 6 independent experiments; 4 weeks old CD4-YY mice treated with doxycycline, n=8 from 4 different experiments, and CD4-YY mice treated with MHC-II blocking antibody, n=3 from 2 different experiments.
Supplemental Figure 4Aii: 20 weeks old CD4-YY mice, n=7 from 3 independent experiments and YYREC/REC TRECherry/Cherry CRE-negative control mice (Ctrl), n=9 from 3 different experiments.
Supplemental Figure 4Aiii: 20 weeks old CD4-YY mice, n=7 from 3 independent experiments.
Supplemental Figure 4Aiv: 20 weeks old CD4-YY mice, n=7 from 3 independent experiments and YYREC/REC TRECherry/Cherry CRE-negative control mice (Ctrl), n=9 from 3 different experiments.
Supplemental Figure 4Bii: 20 weeks old CD4-YY mice, n=7 from 3 independent experiments and YYREC/REC TRECherry/Cherry CRE-negative control mice (Ctrl), n=9 from 3 different experiments.
Supplemental Figure 6B: 1 independent experiment. Thymuses from 8-9 weeks old CD4-YY mice were pooled from n=8 thymuses, TAM-treated Ubiq-YY mice were pooled from n=13 thymuses, and untreated Ctrl mice were pooled from n=12 thymuses.
Supplemental Figure 6D: Thymuses from 20 weeks old CD4-YY thymuses were pooled from n=11 thymuses from 1 independent experiment, and Ctrl mice were pooled from n=18 thymuses from 2 independent experiments.

Randomization

No randomization method was used. Animals were allocated into experiments according to genotype. For experiments involving immortalized MEFs, these lines were generated by our group for their exclusive use in the studies described, so they are no subject to randomization.

Blinding

Animals were allocated into experimental groups according to genotype. Although no specific methods were used for blinding, blood and thymus samples were collected from mice by one individual and then analysed by flow cytometry and scRNAseq by different individuals.

Reporting for specific materials, systems and methods

We require information from authors about some types of materials, experimental systems and methods used in many studies. Here, indicate whether each material, system or method listed is relevant to your study. If you are not sure if a list item applies to your research, read the appropriate section before selecting a response.

Materials & experimental systems

n/a	Involved in the study
	☒ Antibodies
	☒ Eukaryotic cell lines
☒	☐ Palaeontology and archaeology
	☒ Animals and other organisms
☒	☐ Clinical data
☒	☐ Dual use research of concern
☒	☐ Plants

Methods

n/a	Involved in the study
☒	☐ ChIP-seq
	☒ Flow cytometry
☒	☐ MRI-based neuroimaging

Antibodies

Antibodies used

c-Kit-APC/Fire750: clone 2B8, Biolegend, cat # 105838, lot # B381615.
CD34 PE: clone SA376A4, Biolegend, cat # 152204, lot # B378603.
CD135-Biotin: clone A2F10, Biolegend, cat # 135308, lot # B357452.
streptavidin-Brilliant Violet 605: Biolegend, cat # 405229, lot # B387251.
CD45.2- Brilliant Violet 510: clone 104, Biolegend, cat # 109838, lot # B386734.
CD4-PerCP: clone GK1.5, Biolegend, cat # 100432, lot # B357446.
CD8-PerCP: clone 53-6.7, Biolegend, cat # 100732, lot # B329571.
CD127-PE/Cyanine7: clone A7R34, Biolegend, cat # 135014, lot # B395570.
CD19-PerCP: clone 6D5, Biolegend, cat # 115532, lot # B378614.
CD11b-PerCP: clone M1/70, Biolegend, cat # 101230, lot # B399685.
NK1.1-PerCP: clone PK136, Biolegend, cat # 108726, lot # B315703.
TER 119-PerCP: TER-119, Biolegend, cat # 116226, lot # B394121.
CD4-A700: clone RM4-5, Biolegend, cat # 100536, lot # B387950.
CD8-PE/Cyanine 7: clone 53-6.7, Biolegend, cat #, lot # B357446.
CD25- Brilliant Violet 605: clone PC61, Biolegend, cat #102036, lot # B386953.
CD44-APC/Cyanine7: clone IM7, Biolegend, cat # 103028, lot # B399687.
CD24-PE: clone M1/69, Biolegend, cat # 101808, lot # B360234.
TCRbeta- Brilliant Violet 510: clone H57-597, Biolegend, cat # 109234, lot # B367672.
EpCam-PerCPCy5.5: clone G8.8, Biolegend, cat # 118220, lot # B395398.
Ly-51-PE/Cyanine 7: clone 6C3, Biolegend, cat # 108314, lot # B374562.
CD31- Brilliant Violet 711: clone 390, Biolegend, cat # 102449, lot # B389866.
CD34-PE: clone SA376A4, Biolegend, cat # 152204, lot # B378603.
Podoplanin / Gp38-APC Cyanine 7: clone 8.1.1, Biolegend, cat # 127418, lot # B390020.
CD11c-Alexa Fluor 700: clone N418, Biolegend, cat # 117319, lot # B387951.
UEA-1-Biotin: Vector Laboratories, cat # B1065.
CD135-APC: clone A2F10, Biolegend, cat # 135310, lot # B356964.
B220-Alexa Fluor 700: clone RA3-6B2, Biolegend, cat # 103232, lot # B375847.
Sca1- Brilliant Violet 711: clone D7, Biolegend, cat # 108131, lot # B407611.
c-Kit- Brilliant Violet 785: clone 2B8, Biolegend, cat # 105841, lot # B407311.
CD26-APC: clone H194-112, Biolegend, cat # 105841, lot # B407311.
CD127- Brilliant Violet 711: clone A7R34, Biolegend, cat # 135035, lot # B412190.
CD45RB-APC: clone C363-16A, Biolegend, cat # 103320, lot # B414761.
TCRγ/δ-Brilliant Violet 650: clone GL3, Biolegend, cat # 118147, lot # B407692.
CD5-Brilliant Violet 605: clone 53-7.3, Biolegend, cat # 100651, lot # B466751.
CCR4-PE: clone 2G12, Biolegend, cat # 131204, lot # B442558.
CD69-APCCy7: clone H1.2F3, Biolegend, cat # 104526, lot # B444120.
CD62L-Brilliant Violet 650: clone MEL-14, Biolegend, cat # 104453, lot # B467981.
CCR7-Brilliant Violet 785: clone 4B12, Biolegend, cat # 120127, lot # B459147.
TCRbeta-APC: clone H57-597, Biolegend, cat # 109212, lot # B432498.
Anti -GFP rabbit recombinant monoclonal antibody: clone EPR14104, Abcam, cat # AB183734, lot # GR298298-25.
Goat anti-rabbit IgG H&L -Alexa Fluor 555: Abcam, cat # AB150078, lot # GR315964-1.
Goat anti-rabbit IgG H&L -Alexa Fluor 488: Abcam, cat # AB150077, lot # 1034091-13.
Anti-myc tag rabbit monoclonal antibody: clone Vli47, MaineHealth Institute for Research, cat # Vli47, lot # 3.

Validation

c-Kit-APC/Fire750: validated by the manufacturer against C57BL/6 mouse bone marrow cells.
CD34 PE: validated by the manufacturer against C57BL/6 mouse bone marrow cells.
CD135-Biotin: validated by the manufacturer against C57BL/6 mouse bone marrow cells.
CD45.2- Brilliant Violet 510: validated by the manufacturer against C57BL/6 mouse bone marrow cells.
CD4-PerCP: validated by the manufacturer against C57BL/6 mouse splenocytes.
CD8-PerCP: validated by the manufacturer against C57BL/6 mouse thymocytes.
CD127-PE/Cyanine7: validated by the manufacturer against C57BL/6 mouse splenocytes.
CD19-PerCP: validated by the manufacturer against C57BL/6 mouse splenocytes.

CD11b-PerCP: validated by the manufacturer against C57BL/6 mouse bone marrow cells.
NK1.1-PerCP: validated by the manufacturer against C57BL/6 mouse splenocytes.
TER 119-PerCP: validated by the manufacturer against C57BL/6 mouse bone marrow cells.
CD4-A700: validated by the manufacturer against C57BL/6 mouse splenocytes.
CD8-PE/Cyanine 7: validated by the manufacturer against C57BL/6 mouse splenocytes.
CD25- Brilliant Violet 605: validated by the manufacturer against C57BL/6 mouse splenocytes.
CD44-APC/Cyanine7: validated by the manufacturer against C57BL/6 mouse splenocytes.
CD24-PE: validated by the manufacturer against C57BL/6 mouse splenocytes.
TCRbeta- Brilliant Violet 510: validated by the manufacturer against C57BL/6 mouse splenocytes.
EpCam-PerCPCy5.5: validated by the manufacturer against mouse thymic epithelial stromal cell line TE-71.
Ly-51-PE/Cyanine 7: validated by the manufacturer against C57BL/6 mouse bone marrow cells.
CD31- Brilliant Violet 711: validated by the manufacturer against C57BL/6 mouse splenocytes.
CD34-PE: validated by the manufacturer against C57BL/6 mouse bone marrow cells.
Podoplanin / Gp38-APC/Cyanine 7: validated by the manufacturer against mouse thymic epithelial stromal cell line TE-71.
CD11c-Alexa Fluor 700: validated by the manufacturer against C57BL/6 mouse splenocytes.
UEA-1-Biotin: validated by the manufacturer.
CD135-APC: validated by the manufacturer against C57BL/6 mouse bone marrow cells.
B220-Alexa Fluor 700: validated by the manufacturer against C57BL/6 mouse splenocytes.
Sca1- Brilliant Violet 711: validated by the manufacturer against C57BL/6 mouse splenocytes.
CD26-APC: validated by the manufacturer against C57BL/6 mouse bone marrow cells.
c-Kit- Brilliant Violet 785: validated by the manufacturer against C57BL/6 mouse bone marrow cells.
CD127- Brilliant Violet 711: validated by the manufacturer against C57BL/6 mouse splenocytes.
CD45RB-APC: validated by the manufacturer against C57BL/6 mouse splenocytes.
TCRγ/δ- Brilliant Violet 650: validated by the manufacturer against C57BL/6 mouse splenocytes.
CD5-Brilliant Violet 605: validated by the manufacturer against C57BL/6 mouse splenocytes.
CCR4-PE: validated by the manufacturer against hyper-immunized BALB/c splenocytes.
CD69-APCCy7: validated by the manufacturer against PMA+ionomycin-stimulated (6 hours) C57BL/6 mouse splenocytes.
CD62L-Brilliant Violet 650: validated by the manufacturer against C57BL/6 mouse splenocytes.
CCR7-Brilliant Violet 785: validated by the manufacturer against mouse splenocytes.
TCRbeta-APC: validated by the manufacturer against C57BL/6 mouse splenocytes.
Anti -GFP rabbit recombinant monoclonal antibody: clone EPR14104, Abcam, cat # AB183734, lot # GR298298-25.
Goat anti-rabbit IgG H&L -Alexa Fluor 555: validated by the manufacturer for immunocytochemistry/ immunofluorescence against HeLa cells and formalin/PFA-fixed paraffin-embedded sections.
Goat anti-rabbit IgG H&L -Alexa Fluor 488: validated by the manufacturer for immunocytochemistry/ immunofluorescence against rat glial tumor glial cells, mouse embryonic fibroblasts, HeLa cells, MCF7 cells, A431 cells, Jurkat cells and wholemount of Caenorhabditis elegans.
Anti-myc tag rabbit monoclonal antibody: validated by the manufacturer for immunohistochemistry on a paraformaldehyde fixed, paraffin embedded, transgenic mouse mammary tumor expressing a C terminally myc tagged transgene.

Eukaryotic cell lines

Policy information about cell lines and Sex and Gender in Research

Cell line source(s)	The following primary mouse embryonic fibroblast (MEF) lines were generated in our laboratory: E2a-Cre+/Cre; R26-YYGFP/GFP “Yin” sender MEFs, Col1a1-TRE-H2BmCherryCherry/Cherry; R26-YYRec/Rec “Yang” receiver MEFs and TRE-iH2BmGFPGFP/+; R26-YY+/Rec receiver MEFS. Sex of the embryos is unknown.
Authentication	The genotypes for each MEF strain were obtained after isolation of the corresponding embryos by Polymerase Chain reactions (PCRs).
Mycoplasma contamination	The cell lines were no tested for mycoplasm contamination.
Commonly misidentified lines (See ICLAC register)	No cells lines used in this study were found in the database of Commonly misidentified lines.

Animals and other research organisms

Policy information about studies involving animals; ARRIVE guidelines recommended for reporting animal research, and Sex and Gender in Research

Laboratory animals	The study involved the exclusive use of mice (Mus musculus). The following strains were used: Ubiquitin+/ERT2-Cre [B6.Cg-Ndor1Tg (UBC-cre/ERT2)1Ejb/1J, stock #007001]55, R26-Flipase [B6 ROSA26Flpo; B6.129S4-Gt(ROSA)26Sortm2(FLP*)Sor/J, stock #012930]51; E2a-Cre [B6.FVB-Tg(EIIa-cre) C5379Lmgd/J; stock #003724]52, Col1a1-TRE-H2BmCherry [Col1a1-tetO-H2B-mCherry; stock #014602) 53 and CD4-Cre [B6.Cg-Tg(Cd4-cre)1Cwi/BfluJ; stock #022071]56, Yin&Yang mice (R26-YYRec/Rec; Col1a1-TRE-H2BmCherryCherry/Cherry) were generated in our laboratory. Mice were housed in a pathogen-free facility. Both male and female mice at the age of 6–25 weeks were employed in this study.
Wild animals	This study did not involve the use of wild animals.
Reporting on sex	Animals of both sexes were equally used without specifically being selected by sex.

Field-collected samples	This study did not involved samples collected from the field.
Ethics oversight	All experiments involving mice were performed under Queen Mary University of London Veterinary oversight with UK Home Office authorization and comply with all relevant ethical regulations regarding animal research.

Note that full information on the approval of the study protocol must also be provided in the manuscript.

Plants

Seed stocks	*Report on the source of all seed stocks or other plant material used. If applicable, state the seed stock centre and catalogue number. If plant specimens were collected from the field, describe the collection location, date and sampling procedures.*
Novel plant genotypes	*Describe the methods by which all novel plant genotypes were produced. This includes those generated by transgenic approaches, gene editing, chemical/radiation-based mutagenesis and hybridization. For transgenic lines, describe the transformation method, the number of independent lines analyzed and the generation upon which experiments were performed. For gene-edited lines, describe the editor used, the endogenous sequence targeted for editing, the targeting guide RNA sequence (if applicable) and how the editor was applied.*
Authentication	*Describe any authentication procedures for each seed stock used or novel genotype generated. Describe any experiments used to assess the effect of a mutation and, where applicable, how potential secondary effects (e.g. second site T-DNA insertions, mosiacism, off-target gene editing) were examined.*

Flow Cytometry

Plots

Confirm that:

- [x] The axis labels state the marker and fluorochrome used (e.g. CD4-FITC).
- [x] The axis scales are clearly visible. Include numbers along axes only for bottom left plot of group (a 'group' is an analysis of identical markers).
- [x] All plots are contour plots with outliers or pseudocolor plots.
- [x] A numerical value for number of cells or percentage (with statistics) is provided.

Methodology

Sample preparation	Mouse peripheral blood was collected in K2 EDTA-coated capillary tubes (microvette CB300, Sarstedt) from the lateral vein of the tail, lysed in red blood cell lysis buffer (Zen-Bio) and analysed for the expression of GFP. To assess cellular viability, 0.1µg/mL 4',6-diamidino-2-phenylindole (DAPI) staining was used.<br>Immortalized mouse embryonic fibroblasts (MEFs) were derived from E13.5 embryos in our own laboratory. Cells were stained with the corresponding antibodies for 20 minutes at 4ºC, washed with Phosphate buffered saline (PBS) and stained with DAPI to assess live events.<br>Thymuses were collected from euthanised animals and dissociated with a working solution of 1 mg/mL collagenase/dispase (ROCHE) containing 0.1 U/mL of collagenase and 0.8 U/mL of dispase for 30 minutes at 37ºC. After lysis with red blood cell lysis buffer (Zen-Bio), single cell suspensions were stained and analyzed to identify thymic progenitors, thymocytes and thymic stromal cells.
Instrument	Analyses were carried out in both BD Symphony A3 1 equipped with 5 lasers: 355nm Ultra Violet, 405nm Violet, 488nm Blue, 561nm Yellow-Green and 637nm Red and LSR Fortessa 1 equipped with 4 lasers: 405nm Violet, 488nm Blue, 561nm Yellow/green and 640nm Red.
Software	Data was collected with BD FACSDiva Software (version 8.0.1) (BD Biosciences, San Diego, CA) and analyzed with FlowJo version 10.8.0 (BD Life Sciences).
Cell population abundance	Post-sort purity checks were performed to assess the CD45+ and CD45- fractions inside the mCherry+ populations. For this purpose, sorted cells were run to capture a total of 60 events for each one of these populations.

Gating strategy

Single color compensation controls to verify the compensation settings were always acquired along with the samples. 3T3 cells stably expressing mCherry and whole bone marrow cells stained with Gr1 / CD11b coupled with different fluorochromes were employed as single color control. Cells were gated according to their shape and complexity and only singlets and live cells were used for the analysis. Thymuses were stained and analysed to identify early thymic progenitors [ETPs, Lin–(CD11b–CD19–NK1.1–Ter119–)cKit+CD44+CD25–CD24–/+], CD45+B220+ (containing B cells), CD45+Flt3+CD4–CD8–CD25–cKit–/intermediate (containing DC precursors), thymocytes [including single-positive CD4 SP4 cells: CD19–CD11b–NK1.1–TER119–CD8–CD4+; CD25+SP4: CD19–CD11b–NK1.1–TER119–CD8-CD4+CD25high enriched for T regulatory cells; DP cells: CD19–CD11b–NK1.1–TER119–CD8+CD4+; pre-positive selection DPs: CD69–TCRab–/lowCD69–; early post-positive selection DPs: CD69+TCRablow; co-receptor reversing DPs: CD69+TCRabhigh; single-positive CD8 SP8 cells: CD19–CD11b–NK1.1–TER119–CD8+CD4–; immature single positive CD8 ISP cells: CD19-CD11b–NK1.1–TER119–CD8+CD4–CD24+TCRβ–; CD4– CD8– DN cells: CD19–CD11b–NK1.1–TER119–CD8–CD4–; and γδ T cells: CD19–CD11b–NK1.1–TER119– TCRβ–TCRγδ+CD8–CD4–] and thymic stromal cells [including CD45+CD11c+ cells: containing ~75% of DCs and ~25% of macrophages); medullary fibroblasts: CD45–CD31–EPCAM–Gp38+CD26–; capsular fibroblasts: CD45–CD31–EPCAM–CD26+; endothelial cells: CD45–EPCAM–CD31+; CD31+EPCAM+ cells: CD45–CD31+EPCAM+; thymic epithelial cells TEC: CD45–CD31–EPCAM+ and cortical TEC: CD45–CD31–EPCAM+UEA-1–Ly51+].

☒ Tick this box to confirm that a figure exemplifying the gating strategy is provided in the Supplementary Information.
