# Supplementary material for: Unbiased recording and identification of thymic cellular interactomes using synthetic Notch receptors
Source: Nat Commun. 2026 Mar 9;17:3708. doi: 10.1038/s41467-026-70225-5 (PMC13102935; doi:10.1038/s41467-026-70225-5)
Supplement: Supplementary file 4 — Description of Additional Supplementary Files [file 41467_2026_70225_MOESM4_ESM.pdf]

Description of Additional Supplementary Files

File Name: Supplementary Data 1

Description: Top 50 Differentially expressed genes (DEG). DEGs were identified for each cluster using the Wilcoxon rank-sum test with Benjamini-Hochberg p-value correction in Seurat.
